# Tailored Education for Older Patients to Facilitate Engagement in Falls Prevention Strategies after Hospital Discharge—A Pilot Randomized Controlled Trial

**DOI:** 10.1371/journal.pone.0063450

**Published:** 2013-05-23

**Authors:** Anne-Marie Hill, Christopher Etherton-Beer, Terry P. Haines

**Affiliations:** 1 School of Physiotherapy, Institute for Health Research, The University of Notre Dame Australia, Fremantle, Western Australia, Australia; 2 School of Medicine and Pharmacology, Western Australian Centre for Health and Ageing, University of Western Australia, Perth, Western Australia, Australia; 3 School of Physiotherapy, Monash University, Cheltenham, Victoria, Australia; 4 Allied Health Research Unit, Southern Health, Clayton, Victoria, Australia; Tehran University of Medical Sciences, Islamic Republic of Iran

## Abstract

**Background:**

The aims of the study were to evaluate the effect of providing tailored falls prevention education in hospital on: i) engagement in targeted falls prevention behaviors in the month after discharge: ii) patients’ self-perceived risk and knowledge about falls and falls prevention strategies after receiving the education.

**Methods:**

A pilot randomized controlled trial (n = 50): baseline and outcome assessments conducted by blinded researchers. Participants: hospital inpatients 60 years or older, discharged to the community. Participants were randomized into two groups. The intervention was a tailored education package consisting of multimedia falls prevention information with trained health professional follow-up, delivered in addition to usual care. Outcome measures were engagement in falls prevention behaviors in the month after discharge measured at one month after discharge with a structured survey, and participants’ knowledge, confidence and motivation levels before and after receiving the education. The feasibility of providing the intervention was examined and falls outcomes (falls, fall-related injuries) were also collected.

**Results:**

Forty-eight patients (98%) provided follow-up data. The complete package was provided to 21 (84%) intervention group participants. Participants in the intervention group were significantly more likely to plan how to safely restart functional activities [Adjusted odds ratio 3.80, 95% CI (1.07, 13.52), *p* = 0.04] and more likely to complete other targeted behaviors such as completing their own home exercise program [Adjusted odds ratio 2.76, 95% CI (0.72, 10.50), *p* = 0.14] than the control group. The intervention group was significantly more knowledgeable, confident and motivated to engage in falls prevention strategies after receiving the education than the control group. There were 23 falls (n = 5 intervention; n = 18 control) and falls rates were 5.4/1000 patient days (intervention); 18.7/1000 patient days (control).

**Conclusion:**

This tailored education was received positively by older people, resulted in increased engagement in falls prevention strategies after discharge and is feasible to deliver to older hospital patients.

**Trial registration:**

The study was registered with the Australian New Zealand Clinical Trials Registry; ACTRN12611000963921 on 8th November 2011.

## Background

Older people who have been recently discharged from hospital are at high risk of falls and other adverse events [1], [2], [3]. Approximately one third of this population have developed functional decline compared to their pre-admission level of activities of daily living [4], [5]. Up to 40% of patients fall in the first six months after discharge compared with 30% in the general community population [1], [6], [7], [8] and up to 50% of falls during this period result in physical injury [1], [6]. Older people also have over twice the risk of sustaining a hip fracture after a hospital admission, especially in the first four weeks after discharge [9].

Despite this increased risk of falls only a small number of studies have investigated the effect of providing interventions to reduce falls among older people in the post discharge period. A recent randomized controlled trial (RCT) found that in older patients discharged after hip fracture, an extended physiotherapy program which prescribed home exercises to be completed after discharge reduced falls rates by 25%, while cholecalciferol treatment (2000 IU/d) reduced hospital readmissions by 39% [10]. Home visit interventions after discharge that include personalized environmental assessment by a trained health professional and targeted modifications to the physical environment, have also been shown to be effective in reducing falls in high risk groups of older people including those with recurrent falls or hip fracture [11], [12], [13].

When older patients are discharged from hospital to the community there is a transfer in responsibility for health care from the inpatient team to the patient and their community health care team [14], and it is recommended that patients are empowered to take an active role in this transition [15]. However a large observational study that followed older patients for six months after discharge demonstrated that older people have low levels of knowledge about how to reduce their falls risk and low levels of engagement in suitable exercise programs [16], [17]. Risk taking behavior is common among members of this population as a result of patients wanting to test their own physical boundaries, having difficulty recognising and compensating for their own physical limitations and how they change over time, or encountering other barriers to asking for or receiving assistance [18]. Previous recommendations that well-designed falls prevention education be provided to older people [19], [20], [21] thus appear to be particularly applicable to this population.

Recently a large RCT (n = 1206) conducted in a hospital setting evaluated the effect of providing individual patient-level tailored multimedia falls prevention education that was designed using sound pedagogical principles [22], [23]. This intervention reduced falls by approximately 50% in a subgroup of patients with intact cognition, but had no ongoing protective effect in the post discharge period [1]. However no randomized trials have evaluated the effect of providing older hospital patients with tailored falls prevention education that is targeted to the post discharge period on falls rates after discharge.

There were two primary aims of this pilot study: i) to evaluate the effect of providing a tailored multimedia falls prevention education program in hospital prior to discharge and in addition to usual care on engagement in targeted falls prevention behaviors in the month after discharge; ii) to evaluate the effect of the intervention on older patients’ self-perceived risk of falls and knowledge about falls and falls prevention strategies after the education. The secondary aim of the study was to determine older patients’ perceptions of the education program. We also sought to collect data on health outcomes (rate of falls, proportion of people who become fallers and rate of falls related injuries in the first month after hospital discharge) that would be the primary outcomes in a large trial to demonstrate the feasibility of our approach.

## Methods

The protocol for this trial and supporting CONSORT checklist are available as supporting information; see Checklist S1 and Protocol S1.

### Ethical Considerations

Since this was a pilot study, potential participants were informed that the trial was being conducted to test the effect of a novel education intervention, including older peoples’ perceptions of the education. Patients who provided written informed consent were enrolled in the study. The study was approved by The University of Notre Dame Australia and the Sir Charles Gairdner Group human research ethics committees. Trial registration: The study was registered with the Australian New Zealand Clinical Trials Registry; ACTRN12611000963921 on 8^th^ November 2011. https://www.anzctr.org.au/Trial/Registration/TrialReview.aspx?id=343441.

### Design

A two-group pilot randomized trial with blinded baseline and outcome assessment. The design was based on recommended guidelines for conducting pilot studies [24].

### Participants and Setting

Hospital patients who were aged 60 years or older and planned to be discharged from the stroke and rehabilitation units of Swan Kalamunda health service were enrolled in the trial between April 2012 and September 2012 and followed up for one month after discharge. The stroke unit admits patients with a new diagnosis of stroke or patients from other wards or hospitals requiring ongoing stroke rehabilitation. The rehabilitation unit admits older patients undergoing rehabilitation for a variety of geriatric conditions, including fractures, cardiac conditions and general rehabilitation. Patients were eligible to be enrolled in the trial if they spoke English as a first language, could give written informed consent, were to be discharged to the community and had a proposed length of stay in hospital of greater than five days. Patients were not approached to participate in the trial if they were to be discharged to residential care, had hearing or visual problems that prevented them from engaging with education materials or had a Mini Mental State Examination [25] score of less than 24/30.

### Randomization and Blinding

A computer-generated, random number schedule was developed and placed into opaque, consecutively numbered envelopes by a researcher (SM) not involved in the project. The randomization envelopes were stored off the hospital site and one envelope was opened for each participant in order of recruitment on completion of the baseline assessment. The researcher (AMH) telephoned to receive the group allocation number when notified that a patient was enrolled and had received the baseline assessment. The researcher then provided the education intervention on the ward to the participants who were allocated to the intervention group as soon as practicable. Research assistants who approached participants for consent and completed the baseline, discharge and one month follow up assessments were blinded to group allocation. Participants received the intervention privately at their bedside except when in a shared room with another participant when a patient lounge was utilized to minimize contamination. Participants were not informed if they were in the intervention or control group, although participants in the intervention group were made aware that they were receiving education designed to assist them to safely manage at home.

### Intervention

The education intervention design was based on a previously successful program that was tested for pedagogical efficacy [22] and was subsequently found to be effective in reducing falls in hospital in cognitively intact patients when evaluated in a large RCT [23]. Regarding falls prevention it promoted a positive self-identity and emphasized the positive benefits of engagement in post discharge falls prevention strategies [20], [26]. The intervention consisted of providing written and video materials which were designed using adult learning principles [27] and followed recommended guidelines for the presentation of patient education materials [28]. Video materials were viewed by participants using a portable digital video disk (DVD) player with a 9-inch screen and external head phones. This initial session was followed up with individual tailored discussion sessions with the educator at the patient’s bedside and a single telephone call two weeks after discharge to reinforce the education. One-to-one follow-up reinforcement in hospital was designed to be completed in two sessions of approximately 15 minutes, but the number of actual sessions varied between two and five depending on how long participants required to discuss each section of the workbook and whether there were any interruptions to the session. Information presented was based upon local data and data presented in previous research [1], [6], [18], [21] and emphasized developing personalized behavioral strategies to maintain safety (i.e. prevent falls) while regaining function after hospitalisation. Key messages focused on: i) seeking assistance for functional activities; ii) gradually resuming functional activities; iii) planning to participate in an exercise program. The content and progression of the education was based on the Health-Belief Model [29] and informed participants of the risk of falls and functional decline after discharge and about falls prevention strategies that they could undertake in the period after discharge. The program identified barriers and facilitators to undertaking such strategies, fostered patient belief that they could successfully undertake such strategies and that if undertaken, their risk of falling would reduce, and provided cues for action thus facilitating patient planning to undertake these strategies. The educator facilitated the development of specific personalized strategies which participants were assisted to write in their workbook. These were revised, and if required updated, by the participant during the follow-up telephone call. This was a tailored behavior change model of education, where participants were educated to develop the capability and the motivation to undertake their strategies when the opportunity presented in their home situation [30], [31]. The educator (AMH) was a physiotherapist, who was previously trained in delivering patient falls prevention education and had post-graduate educational qualifications.

### Control Conditions

All participants continued to receive their usual care in preparation for discharge. Usual care in the setting was provided by a multidisciplinary team and included 24 hour medical and nursing care, physiotherapy and occupational therapy five days per week and local ward programs of falls risk assessment and management. To prepare for discharge there was a “hospital in the home” program directed by the multi-disciplinary team who determined which patients would be offered specific services. These services included home visiting staff for personal care or nursing care and in home occupational and physiotherapy up to four times per week to aid rehabilitation if required. Social work services for the participant and their family and a discharge letter to the participant’s community doctor were also provided. Participants were also referred to outpatient services as deemed required, for example for ongoing physiotherapy exercises or a review of their condition by the geriatric outpatient care team.

### Outcome Measures

The primary outcome measures were participants’ engagement in falls prevention strategies in the month after discharge and participants’ self-perceived risk and knowledge about falls and falls prevention strategies to engage in after hospital discharge. Secondary outcomes were participants’ knowledge gain and perceptions of receiving the education. The number of falls and falls injuries sustained by participants in the month after discharge was also measured. These outcome measures were categorized based on Kirkpatrick’s four-level model of evaluating training programs [32]. These are: level 1, reaction (older patients’ perceptions of receiving the education); level 2, learning (knowledge gain from receiving the education); level 3, behavior change after the education (engagement in falls prevention strategies); level 4, resultant outcomes (health outcomes of preventing falls and falls injuries).

#### Level 1 and Level 2 - Knowledge gain and perceptions of the education program

Participants’ knowledge of falls epidemiology was measured at baseline and after receiving the education. Participants were also surveyed at baseline and immediately prior to discharge to evaluate their self-perceived risk of falls and falls injuries. The perception to receiving the education in the intervention group was measured by evaluating participants’ self-perceived risk of falls and falls injuries and their confidence and motivation to engage in falls prevention strategies before and immediately after receiving the education and one month after the education. These outcomes were measured with surveys which were modified from previously tested surveys designed to evaluate patient falls prevention education [22]. Survey item responses used a Likert scale (strongly agree; agree; undecided; disagree; strongly disagree) except for knowledge items where a “desired” response was determined based on the content of the education.

#### Level 3 - Health behaviors

Health behaviors were the number of falls prevention strategies engaged in by participants in the first month after hospital discharge. Falls prevention strategies that were facilitated by receiving the education were grouped into three categories. The first category was seeking formal (care agency) or informal (family or friends) assistance with activities of daily living (ADL) or instrumental activities of daily living (IADL). ADL were defined as receiving assistance with eating, bathing, toileting, dressing, transferring or walking [33] and IADL were defined receiving assistance with handling finances, housework, meal preparation, medication, transport, telephoning or shopping [34]. The second category was planning to gradually return to independently undertaking usual functional activities by planning which aspects of the activity might require assistance from another person, completing a gradual implementation of the activity and informally modifying the home environment to allow the activity to be completed safely. Informally modifying the home environment was categorized as either independently or with family assistance removing clutter, altering home layout to allow activities to be completed safely or using aids and appliances to assist in completing activities. The third category was participating in an exercise program at least once per week, defined as a multiple component (containing strength and balance) exercise program [35]. The exercise could be completed as a group or home program supervised by a health professional, an independent home program (excluding walking only) or another type of formal exercise program, including dancing or tai chi. These outcomes were measured by conducting a baseline face to face structured interview in hospital to establish participants’ current levels of engagement in these strategies and then conducting a structured telephone interview with each participant at one month post discharge, during which participants were asked about their levels of engagement of each strategy after discharge. Response options were Yes; No (participant did or did not engage in that particular strategy).

#### Level 4 - Health outcomes

Health outcome measures were the number of falls and falls injuries sustained by each participant in the first month after hospital discharge. The definition of a fall event was the World Health Organization definition namely: “an event which results in a person coming to rest inadvertently on the ground or floor or other lower level” [36]. A fall event was classified as an injurious if an injury was reported by a participant following a fall. Injuries were classified as none reported, bruise, pain, laceration, dislocation or fracture. Falls and falls related injuries were measured using a diary issued to each participant at time of discharge with instruction in its use, with a subsequent telephone call at one month post discharge.

Other outcomes were measured to evaluate the program feasibility and included the number and content of education sessions delivered, the number of strategies identified by participants in the intervention group after receiving the education, and the number of participants who identified receiving falls prevention education during their participation in the study.

Other interventions provided by health care professionals that were not included in the education program but could potentially affect rates of falls by participants after discharge were also measured. These variables were based on the current evidence based practice for falls prevention in the community, in particular in the post discharge period [35], [37]. These were a visit by the hospital occupational therapist at or in the first month after discharge, attending a falls clinic for assessment and management, receiving a vision intervention, such as new glasses, or receiving a medication review, including withdrawal of psychotropic medication by the participant’s medical provider.

Other measures collected at enrolment included age, diagnosis, length of stay in hospital, falls history, education level attained (primary, grade 10, grade 12, technical college university), community living situation (home alone, home with partner, home with other) health- related quality of life measured using the EQ-5D [38], visual impairment, self- reported use of four or more medications, self-reported use of psychoactive medications and self-report of depressed mood.

### Procedure

Patients who met inclusion criteria were approached and informed both verbally and in writing about the study and those who provided written informed consent were enrolled. Recruitment occurred between April 2012 and September 2012. Research assistants completed baseline assessments, then participants were randomized into either the intervention group or the control group. Both groups continued to receive their usual care and, in addition, the intervention group received the education intervention in a pragmatic manner, most usually on three or four consecutive days. The educator used a patient lounge to provide the education sessions if participants were in a shared room with another participant. The survey that measured the effects of the education was administered by the educator when the intervention was completed, to evaluate the effect of the education on raising participants’ levels of awareness, knowledge, confidence and motivation regarding engaging in falls prevention strategies after discharge. In the 24 hours prior to discharge research assistants completed discharge assessments and issued participants with a falls diary and instruction in its use.

At one month post discharge participants were contacted by research assistants who used recommended questioning [39] to ascertain if the participant had fallen and if they had sustained any falls-related injuries. Subsequently the research assistant administered the one month survey to evaluate levels of engagement in falls prevention strategies. After the survey, additional survey items were administered to the intervention group that evaluated their knowledge, confidence and motivation regarding engaging in falls prevention strategies one month after receiving the education. Research assistants were physiotherapists who were experienced in working with older people and knowledgeable regarding local discharge procedures and other local community programs provided for older people. Finally, after completing the survey, participants were given information about local falls prevention programs and if required, assistance to contact the relevant providers.

### Statistical Analysis

All analyses were conducted on an intention to treat principle. Two participants (one in the intervention group and one in the control group) who did not provide any follow up data were treated as missing. Utility scores were constructed for the EQ-5D scores using the Dolan formula [40]. Alpha level for significance was set to *p*<0.05 for all comparisons. All analyses were conducted using Stata 11 software (StataCorp, 2009. Stata Statistical Software: Release 11. College Station, TX: StataCorp LP. StataCorp, Texas).

Participants’ levels of self- perceived risk of falls and falls injuries and knowledge of falls epidemiology before and after receiving the education were compared between groups using logistic regression. The participants in the intervention group self-perceived risk of falls and confidence and motivation to engage in falls prevention strategies before and immediately after receiving the education was compared using Wilcoxon signed-rank tests. Participants’ levels of awareness about and perceptions to receiving the education were summarized using descriptive statistics (number, percentage).

The differences between groups of the proportions of participants engaging in falls prevention strategies (health behaviors) after discharge was evaluated using logistic regression with adjustment for length of observation after discharge and baseline values of these variables, and results presented as adjusted odds ratios (AOR) with 95% confidence intervals (CI). Post estimation of goodness of fit of the models was calculated using the Hosmer-Lemeshow test [41] and the area under the receiver operating characteristic curve.

Falls rates (health outcomes) in the month after discharge were analyzed using negative binomial regression with 95% CI, adjusted for participants’ length of observation after discharge and results reported as adjusted incident rate ratios. The proportion of participants having one or more falls in each group (being a faller) was compared between groups using logistic regression, with adjustment for length of observation after discharge and results reported as AOR.

### Sample Size

The sample size for the pilot study was determined as n = 50. This was not a formal calculation of statistical power for testing the intervention for its effect on falls outcomes, but was based on determining the feasibility of testing this novel intervention for its effect on rates of falls in a similarly designed larger study [24]. Key measures of interest were determining whether intervention group participants would have a positive perception (increased confidence and motivation levels) of the education, whether providing the education in hospital would have the desired effect of raising participants’ levels of knowledge and subsequent engagement in falls prevention strategies at home after discharge, whether health outcomes (falls) could be collected concurrently and whether the measurement methods chosen would detect the effect of the intervention on engagement in falls prevention strategies. The time and number of sessions required to deliver the education was also of interest to establish the feasibility of providing this type of intervention in a hospital setting. The one month period was chosen to give participants sufficient time to engage in their chosen strategies after discharge.

## Results

### Participant Characteristics

**Figure 1:**
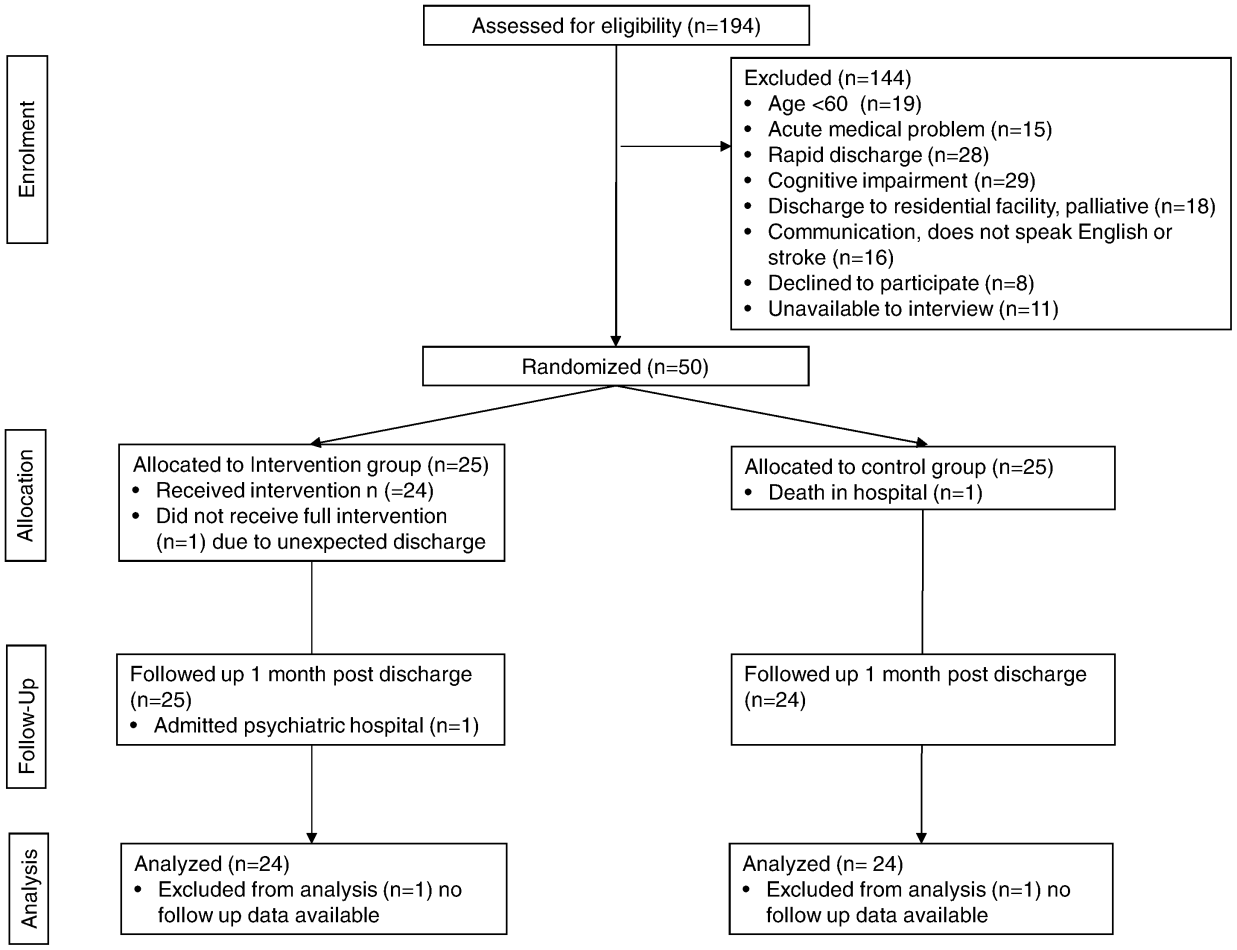
Participant flow through study.

There were 50 patients enrolled in the trial. One participant in the control group died in hospital after being recruited but before being discharged and one participant in the intervention group self-discharged from hospital prior to receiving the complete intervention and was not contactable following discharge. This participant was subsequently admitted to a psychiatric ward in the month after discharge and was medically assessed as too unwell to be interviewed. The demographic characteristics of the participants are presented in Table 1 and the flow of participants through the study is presented in Figure 1.

**Table 1:** Characteristics of participants at point of enrolment into study.

Variable	Intervention (n = 25)	Control (n = 25)
Age, (years) mean ±SD	78.2±9.0	78.3±7.5
Female n (%)	16 (64.0)	17 (68.0)
**Diagnosis n (%)**		
Stroke	5 (20.0)	7 (28.0)
Other neurological	1 (4.0)	3 (12.0)
Orthopedic	8 (32.0)	1 (4.0)
Cardiac or pulmonary	4 (16.0)	8 (32.0)
Other geriatric management or other surgery	7 (28.0)	6 (24.0)
Length of stay in hospital (days)	32.4±42.0	31.2±34.5
Hospital admission in 6 months prior to current admission n (%)	10 (40.0)	1 (4.0)
Faller in 6 months prior to hospital admission n (%)	13 (52.0)	10 (40.0)
Faller during hospital admission n (%)	3 (12.0)	2 (8.0)
Visual impairment^a^ n (%)	9 (36.0)	12 (48.0)
**Discharge destination n (%)**		
Home alone	10 (40.0)	3 (12.0)
Home with partner	12 (48.0)	12 (48.0)
Home with other	2 (8.0)	7 (28.0)
Other^b^	1 (4.0)	3 (12.0)
**Discharge mobility n (%)**		
No aid	6 (24.00)	8 (32.0)
Walking stick	3 (12.0)	4 (16.0)
Walking frame	14 (56.0)	11 (44.0)
Wheelchair	2 (8.0)	1 (4.0)
Unable to mobilize without assistance		1 (4.0)
**Health related quality of life at discharge**		
EQ-5D^c^ –utility, mean±SD	0.5±0.3	0.6±0.3
EQ-5D^d^ –VAS, mean+SD	69.0±16.2	65.7±16.8
**Highest education level attained n (%)**		
Primary	11 (44.0)	10 (40.0)
Grade 10	11 (44.0)	4 (16.0)
Grade 12	2 (8.0)	6 (24.0)
Technical college	0 (0.0)	3 (12.0)
University	1 (4.0)	2 (8.0)
Self-report taking 4 or more medications n (%)	21 (84.0)	18 (72.0)
Self-report diagnosis of depression n (%)	4 (16.0)	4 (16.0)
Self-report taking psychoactive medications^e^ n (%)	12 (48.0)	7 (28.0)

a cataracts(untreated), macular degeneration, glaucoma.

b transitional care facility, death.

c Euro qol Dolan method, range −0.59 to 1.0 higher indicates better self-perceived health-related quality of life.

d Euro qol visual analogue scale, range 0–100 higher indicates better self-perceived health-related quality of life.

e Includes anti-psychotic, anti- depressant, mood stabilizing medication.

### Intervention Delivery

The inpatient intervention (DVD, workbook and follow up sessions) was delivered to 25 participants in the intervention group. One participant received the workbook only and three participants received only two sessions and did not complete the entire education as intended. No control group participants received the education. The median (interquartile range) number of follow-up sessions provided in hospital was 3 (3, 4), 21 (84%) participants were assisted to complete a written plan in their workbook and 21 (84%) of participants received a follow up telephone call. The median (interquartile range) time to deliver the education, including the DVD and follow up telephone call was 45 (35.5, 55) minutes. Participants in the intervention group identified a total of 98 behavioral strategies that they planned to engage in after discharge, with a median (interquartile range) of 4 (3.5, 5) strategies identified by each participant. Participants also identified 36 potential barriers that could prevent them from performing their strategies with a median (interquartile range) of 2 (1, 2) barriers identified by each participant.

There were no adverse events attributable to the education intervention or to taking part in the study.

### Knowledge Gain and Perception of Receiving the Education Program

Baseline levels of knowledge of falls epidemiology were not significantly different between the groups with 8 (33.4%) intervention and 12 (50.0%) control group participants giving a desired response to survey item 11: “For every 100 patients who leave the hospital, how many do you think would fall over in the community after discharge” and 8 (33.4%) intervention and 6 (25.0%) control group participants giving a desired response to survey item 12: “For every 100 falls that occur when a person goes home from hospital, how many do you think would result in a physical injury, such as a bruise, a cut, a head injury, or even a broken bone.” When surveyed prior to discharge and after receiving the education, more intervention group participants were able to give a desired response to survey item 11 [intervention n = 18 (75.0%), control n = 12 (50.0%), Odds ratio 3.0, 95% CI (0.88, 10.18), *p* = 0.08]. Significantly more participants in the intervention group were able to give a desired response to survey item 12 [intervention, n = 19 (79.2%), control n = 6 (25.0%), Odds ratio 9.22, 95% CI (2.46, 34.58) *p* = 0.001]. There were no significant differences between the groups in participants’ self-perceived risk of falls and falls injuries when surveyed at baseline. Participants in the intervention group had a significantly increased self-perceived risk of falls [OR 4.96, 95% CI (2.84, 7.10), *p*<0.001] and of falls injuries [OR 4.76, 95% CI (2.59, 6.94), *p*<0.001] compared to the control group when surveyed prior to discharge and after receiving the education.

The intervention group was surveyed immediately after completing the education and their perceptions to receiving the intervention is presented in Table 2. Participants in the intervention group had significantly increased self-perceived risk of falls and falls injuries, increased self-awareness of falls prevention strategies that they could use after discharge and were more confident and motivated to engage in falls prevention strategies than before they received the education. When participants in the intervention group were surveyed at one month post discharge to determine their perceptions to receiving the education, 21 (87.5%) participants strongly agreed or agreed that their knowledge levels had increased by receiving the education. Eighteen (75.0%) participants strongly agreed or agreed that the education had made them more confident to reduce their falls risk and regain their independence at home, 21 (87.5%) participants strongly agreed or agreed that they were motivated to continue their falls prevention activities and 22 (91.7%) participants strongly agreed or agreed that the education should be provided to other older people at discharge. When asked “Did you receive education from the researcher (AMH) in hospital and a phone call after discharge?”(response option: Yes, No) 23 (95.83%) participants in the intervention and 2 (8.33%) participants in the control group responded “Yes.”

**Table 2:** Participants’ perceptions (awareness, knowledge gain, confidence and motivation) of receiving education.

Item	Item wording	Response (n = 25 baseline, n = 22 after education)^a^					
		SA^b^	A	U	D	SD	p-value
1	I think that older people are at risk of falling over during the 6 months after hospital discharge.”						
	Baseline	4 (16.0)	16 (64.0)	4 (16.0)	1 (4.0)		
	After education	19 (86.4)	3 (13.6)				<0.001
2	I think that I would be likely to fall over during the next 6 months						
	Baseline	1 (4.0)	12 (48.0)	8 (32.0)	4 (16.0)		
	After education	18 (81.8)	3 (13.6)	1 (4.6)			<0.001
3	I think that if I were to fall over in the next 6 months, I would be likely to get an injury (for example, a cut, a bruiseor even a broken bone).						
	Baseline	7 (25.0)	12 (50.0)	3 (12.5)	3 (12.5)		
	After education	18 (81.8)	4 (18.2)				0.001
4	I think that I could be at risk of decreased independence in the first 6 months after I return home						
	Baseline	1 (4.0)	14 (56.0)	5 (20.0)	5 (20.0)		
	After education	15 (68.2)	3 (13.6)	4 (18.2)			<0.001
5	I feel I am now more aware of the problem of falls after discharge from hospital	16 (72.7)	5 (22.7)	1 (4.6)			
6	The education has made me more motivated to improve my safety and regain my independence than what I wasbefore I participated in the education	17 (77.3)	4 (18.2)	1 (4.5)			
7	I feel I am now more aware of strategies that I can use and improve my safety and regain my independence afterdischarge	19 (86.4)	2 (9.1)	1 (4.5)			
8	I am confident that I could attempt these strategies (referring to participant’s chosen strategies)	13 (59.1)	5 (22.7)	4 (18.2)			
9	I am very motivated to improve my safety and independence at home in the first month after discharge by usingthese strategies	18 (81.8)	4 (18.2)				

a Three participants did not complete post education survey.

b Likert scale where SA = strongly agree, A = agree, U = undecided, D = disagree, SD = strongly disagree.

### Health Behaviors

Participants’ engagement in falls prevention strategies that were the target of the education intervention are presented in Table 3. Participants in the intervention group were more likely to seek formal assistance for ADL [AOR 3.02, 95% CI (0.82, 11.10) *p* = 0.09] and IADL [AOR 2.53, 95% CI (0.75, 8.59), *p* = 0.14], plan to gradually resume functional activities [AOR 3.80, 95% CI (1.07, 13.52), *p* = 0.04], participate in their own home exercise program [AOR 2.76, 95% CI (0.72, 10.50), *p* = 0.14] and make their own informal home modifications [AOR 2.43, 95% CI (0.74, 7.96), *p* = 0.14], although only one comparison reached statistical significance.

**Table 3:** Participants’ engagement in falls prevention strategies facilitated by education.

Falls prevention strategies	Intervention n = 24 (100%)		Control n = 24 (100%)		Adjusted odds ratio, (95% confidence interval), *p*-value^a^	Model fit (goodness of fit *p*-value/area under the ROC)
	Baseline	One month after discharge	Baseline	One month after discharge		
**Assistance with ADL** b						
Formal services	2 (8.3)	12 (50.0)	1 (4.2)	6 (25.0)	3.02, (0.82, 11.10), 0.09	0.25/0.65
Informal services^c^	2 (8.3)	5 (20.8)	2 (8.3)	9 (37.5)	0.40, (0.10, 1.57), 0.19	0.36/0.68
**Assistance with IADL** d						
Formal services	7 (21.2)	14 (58.3)	4 (16.7)	8 (33.3)	2.53, (0.75, 8.59), 0.14	0.45/0.69
Informal services	6 (25.0)	17 (70.8)	4 (16.7)	13 (54.2)	1.90, (0.54, 6.73), 0.32	0.15/0.60
**Exercises**						
Own home program^e^	1 (4.2)	18 (75.0)	6 (25.0)	11 (45.8)	2.76, (0.72, 10.50), 0.14	0.36/0.73
Formal program^f^	5 (20.8)	13 (54.2)	5 (20.8)	16 (66.7)	0.58, (0.17, 1.93), 0.37	0.20/0.66
Informal home modifications^g^	11 (45.8)	13 (54.2)	10 (41.7)	8 (33.3)	2.43, (0.74, 7.96), 0.14	0.30/0.65
**Gradual return to functional activity**						
Plan (informal modifications, assistance, activity graduation)		18 (75.0)		8 (33.3)	3.80, (1.07, 13.52), 0.04	0.39/0.70

a Adjusted for levels of engagement prior to intervention and length of time of observation after discharge.

b Activities of daily living.

c Assistance from family, friends or others.

d Instrumental activities of daily living.

e Includes program originally designed by health care professional or designed by participant themselves.

f Includes program provided by health care professional either in the home, at a centre or outpatient setting.

g Includes remove clutter, alter layout for easy access, use aids and appliances.

There were no significant differences between the groups in other interventions provided by health care professionals that were not included in the education, but could potentially affect rates of falls by participants after discharge (home visit from hospital occupational therapist, attendance at falls clinic, vision intervention or medication review by participant’s medical provider).

### Health Outcomes

Fall and falls related injuries reported for the one month after discharge are presented in Table 4. Falls data were collected from 48 participants with 2(4.0%) participants not providing any data. There were 23 reported falls, five in the intervention group and 18 in the control group. Three participants sustained fractures; two in the control group (neck of femur, ribs) and one in the intervention group (pelvis). The falls rate in the intervention group was 5.4 falls/1000 patient days and the falls rate in the control group was 18.7 falls/1000 patient days.

**Table 4:** Falls outcomes after hospital discharge.

	Intervention (n = 24)	Control (n = 24)	Adjusted incident rate ratio, 95% CI, *p*-value/Adjusted odds ratio, 95% CI, *p*-value
Falls,/injurious falls/fallers/fractures, days ofobservation after discharge, n	5/2/4/1/953	18/10/9/2/962	
Falls, rate/1000 person days	5.4/1000	18.7/1000	3.38, (0.98, 11.56), 0.05
Injurious falls, rate/1000 person days	2.2/1000	10.4/1000	4.42, (0.66, 29.54), 0.12
Fallers, % group having one or more falls	16.7	37.5	3.02, (0.77, 11.80), 0.11
Number of participants with one or more hospital admissions or doctors’ visits, n (%)	2 (8.3)	4 (16.7)	2.21, (0.36, 13.39), 0.40

## Discussion

This is the first randomized trial to provide a falls prevention multimedia education package with tailored individual follow up for older people at point of hospital discharge. Our study has shown that it is possible to successfully provide education of this nature to a high proportion of participants who enrolled in the study, despite the busy and unpredictable ward environment. Participants were positive in their reaction to the tailored education format [32] which contrasts strongly with previous qualitative studies which reported that older people perceive that falls prevention education is confusing or patronizing [42], [43]. Our results also suggest that participants who received the education developed the capability and motivation to engage in falls prevention behaviors [31]. This is important when designing and evaluating falls prevention education interventions as previous studies have found that older people do not view themselves as personally susceptible to falls [43], have poor levels of knowledge about falls and falls prevention [19], [44] and have low levels of intention to engage in falls prevention programs [45]. A recent national survey found that over 60% of older people are not willing to participate in any type of program to manage concerns about falls [46].

While all participants engaged in some falls prevention strategies before the intervention and after discharge, more participants in the intervention group engaged in strategies that reflected the three key education messages (seek required assistance for ADL and IADL, engage in exercise and plan a graduated return to usual functional activities) during the month after discharge. A key theme of our education was planning a gradual return to functional activities and participants who received the education were significantly more likely to actively plan how to safely re-start a functional activity. Although older people often decline functionally during hospitalization [47], [48] they may purposefully engage in ADL after discharge with the aim of reducing the risk of losing their independence and autonomy, despite the physical risk of falling associated with completing these activities [18], [21]. Alternatively older people may cope with the risk of falls in a suboptimal manner by restricting their engagement in ADL [49], [50].

The positive effect of the intervention in raising participants’ engagement in falls prevention strategies and the positive response of participants to the program, combined with the finding that our approach for collecting data on health behaviors and health outcomes was feasible is a promising result arising from this pilot study. It indicates a larger trial that is adequately powered to detect changes in health outcomes (falls and falls related injuries) is warranted. Although this was a novel intervention we previously demonstrated that this type of positive tailored behavior change program could reduce falls in cognitively intact older hospital patients [23]. A previous study demonstrated that inpatient training regarding participating in a home exercise program reduced falls after discharge in hip fracture patients [10], however a recent review of falls prevention interventions found that there was no conclusive evidence that education could reduce falls in community dwelling older people [35]. Systematic reviews of interventions provided at discharge that aim to improve post discharge outcomes have concluded that there is some evidence that education interventions provided both in hospital and after discharge may have positive effects and should be further tested to determine their impact on health outcomes [3], [51].

Our procedure on the ward for delivering education only to participants in the intervention group appeared successful although two participants in the control group stated that they had received education. This could have due to been participating in the surveys or due to usual care activities of hospital staff members, as we accounted for each workbook issued and were confident that control group participants were not in the room when the DVD was viewed by intervention group participants. Additionally, no control group participant received personal discussion from the educator or a telephone call. Participants in the control group were not specifically informed that they were not receiving education and received all baseline and outcome assessments as well as multidisciplinary team information that was provided to patients on the wards. Social interaction could be an important factor mediating the effect of the intervention and a larger trial could use an active control group with social visits.

There were limitations to conducting the pilot trial. It could be that the intervention group were slower to recover functional ability than the control group by engaging in their chosen falls prevention strategies. Participants may have reduced their independence to avoid falling during the month of observation and may have fallen in subsequent months as they continued to increase their ADL. In a larger trial functional ability should be measured at baseline and at regular intervals until the conclusion of the trial. A larger sample size is required to directly determine the effect of the intervention on falls rates. Falls rates should also be measured for a longer period of time to evaluate whether the decrease in falls is sustained for longer than one month. Additionally while participants were positive in their reaction to the education we were unable to measure health-related quality of life at the conclusion of the trial and this should be included in the larger trial. Our sample size did not allow us to differentiate the effect of the education on subgroups where the effect may be modified. We sought to enrol patients diagnosed with a broad range of medical conditions and our results indicate that it is feasible to tailor the education appropriately, but each group contained small numbers. Similarly social support such as whether participants live alone, could also affect the participants’ capability, motivation and opportunity to engage in falls prevention strategies [17], [31], [52].

In conclusion older patients who have recently been discharged from hospital are at increased risk of falls in the post discharge period. A novel tailored education program was received positively by older people and facilitated engagement in falls prevention strategies after discharge. This pilot study demonstrates that it is feasible to test this intervention in a larger trial to evaluate its effect on falls and falls-related injuries in this population.

## Supporting Information

Checklist S1 **CONSORT checklist.**
(PDF)

Protocol S1 **Trial protocol.**
(PDF)
