# Supplementary material for: Tailored Education for Older Patients to Facilitate Engagement in Falls Prevention Strategies after Hospital Discharge—A Pilot Randomized Controlled Trial
Source: PLoS One. 2013 May 23;8(5):e63450. doi: 10.1371/journal.pone.0063450 (PMC3662677; doi:10.1371/journal.pone.0063450)
Supplement: Protocol S1 — Trial protocol. (PDF) [file pone.0063450.s002.pdf]

Title: The effect of providing tailored multimedia patient education on older patients' engagement in falls prevention strategies after hospital discharge – a randomised controlled trial **ANZTCTR number**: ACTRN12611000963921

Lay title: Does patient education prevent falls after patients are discharged from hospital?

Grant funding: The Menzies Foundation (administered by The University of Notre Dame Australia Research and Finance Offices) Email: research@nd.edu.au

Principal researcher: Dr. Anne-Marie Hill

The University of Notre Dame Australia

Building 46 / Room 407,

School of Physiotherapy, Fremantle Campus

19 Mouat St (Box 1225)

Fremantle WA 6959

Email: Anne-Marie.Hill@nd.edu.au

Ph: +61 8 9433 0239

Fax: +61 8 9433 0210

Associate researcher: Associate Professor Christopher Beer

University Western Australia, HDWA (Aged Care Services, SKHS)

Email: Christopher.beer@health.wa.gov.au

Contact person: Dr. Anne-Marie Hill (as above)

Protocol Summary

**Title**	The effect of providing tailored multimedia education in addition to usual care compared with usual care alone on older patients' engagement in falls prevention strategies after hospital discharge – a randomised controlled trial
**Short Title**	The effect of providing tailored multimedia patient education on older patients' engagement in falls prevention strategies after hospital discharge – a randomised controlled trial
**Protocol Number**	ACTRN12611000963921
**Design**	Clinical trial – randomised controlled trial
**Methodology**	Single blinded trial (blinded baseline and outcome assessors)
**Study Duration**	Estimated recruitment and 1 month follow up of all participants: 1st January 2012 to 31st April 2012
**Study Centre**	Swan Districts Hospital, Midland WA
**Objectives**	Evaluate the effect of providing a tailored multimedia falls prevention education program on:<br>a) self perceived risk of falls, knowledge about falls prevention strategies, confidence and motivation to engage in falls prevention strategies in the first month after hospital discharge<br>b) levels of engagement in falls prevention strategies in the first month after hospital discharge
**Number of Subjects**	50
**Diagnosis and Main Inclusion Criteria**	Patients 60 years and older, admitted to medical or rehabilitation wards, within 1 week of discharge from hospital, no history of dementia, able to give informed consent, discharge to community dwelling
**Intervention**	Individual patient level multimedia education package with trained health professional follow up in addition to usual care
**Duration of intervention**	3 to 4 sessions in hospital (2-4 days) with 1 telephone call to patient at home after discharge
**Control**	Continue to receive usual care
**Main Outcome measure**	Number of falls prevention strategies engaged in after discharge measured by telephone survey
**Statistical Methodology**	Wilcoxon rank sum tests to compare outcomes with ordinal scaling, differences between groups measured using logistic regression.

	Index	Page no
**1**	Study Timeline	4
**2**	Introduction and Background	5
**3**	Aims	7
**4**	Design	8
**5**	Participants and Setting	9
	Inclusion / Exclusion criteria	10
**6**	Outcome measures	12
**7**	Intervention	14
**8**	Statistical Analysis	16
**9**	Safety and Adverse Events	18
**10**	Procedures	20
	Recruitment	20
	Randomisation and Blinding	21
**11**	Data handling and storage	23
**12**	Monitoring	24
**13**	Ethical considerations	25
**14**	Budget	26
**15**	References	28

1. Study Timeline

	Date
Ethics approval (Notre Dame, SKHS)	October 2011
Print materials, employ research assistant, training	November 2011 – December 2011
Site meetings, orientation	January 2012
Recruitment for RCT - enrol participants , data collection, provide intervention in hospital	January 2012 – March 2012
Data collection - follow participants for 1 month after discharge	January 2012 – April 2012
Data management	April 2012 – June 2012
6 monthly report	May 2012
Statistical analyses	June 2012 – August 2012
Preparation of manuscripts, dissemination of results	September 2012 – November 2012
Evaluation of project	December 2012
Final reports	December 2012

2. Introduction and Background

Older people are at increased risk of falls after hospital discharge and their risk of hip fracture during the post discharge period is substantially increased.[1, 2] Older patients who have been discharged from hospital are also at risk of other adverse events such as functional decline, infection, medication complications, unplanned readmissions to hospital and other social and emotional problems.[3-5] Recently we conducted an observational follow up to an RCT (n=343) at Swan Districts hospital.[1] The data from this trial are the first to demonstrate that falls in a general post discharge population are substantially higher than in the general community with over 40% of participants falling at least once in 6 months and over 50% sustaining and injury compared to the yearly rate of falls (30%) and injury (10%) reported in the general community-dwelling older population.

Despite this increased risk of falls only a small number of RCTs have investigated the effect of providing interventions to reduce falls by older people in the post discharge period. Some interventions provided once a patient has left hospital, such as home visits by an occupational therapist and an exercise intervention commenced in hospital that included home training instructions have been shown to reduce falls in high risk populations.[6,7] Meta-analyses have concluded that there is inconclusive evidence about the effectiveness of interventions such as follow up home visits and intensive discharge planning in reducing falls and other adverse events such as hospital readmission.[5]

When patients are discharged from hospital they are expected to assume responsibility for their own health care, including falls prevention, yet no randomised trials have tested falls prevention patient education as an intervention for this at-risk population. Previous observational studies conducted in community populations have identified that older people dismiss falls prevention education as not personally relevant and suggest barriers to engaging in falls prevention activities.[8] Our data from two large observational studies of patients discharged from Swan Districts hospital demonstrate that older people have low levels of knowledge about how to reduce their falls risk and low levels of engagement in suitable exercise programs in the six months after hospital discharge.[9,10] Our recent data

from a successful RCT (n=1206) also demonstrate that in-hospital falls are reduced by approximately 50% in patients with intact cognition by providing individual patient level tailored multimedia education that is designed using sound pedagogical principles.[11] These data when viewed together, suggest that modifying this education program to focus on the post discharge period and delivering it to older patients at point of discharge is a viable means to reduce falls after discharge.

Falls are the leading cause of injury-related hospital admissions in Australia[12] and falls worldwide are a substantial economic burden with an estimated cost of between 0.85 and 1.5% of total health care expenditure in developed countries.[13] Specific to post discharge populations, large investigations have reported rates of adverse events of between 20% and 23%[4] with unplanned hospital readmissions in the US estimated to cost approximately $8 billion.[14] Consequently researchers have concluded that patient safety has been neglected in this area and that adverse events such as falls urgently require further investigation. This study will be the first RCT conducted to investigate the effect of providing multimedia patient education to reduce falls in this older population.

---

3. Aims

3.1 Research Questions

1. Will providing a tailored individual education program for older patients that focuses on falls prevention after discharge in addition to usual care affect older patient's self perceived risk of falls, knowledge about falls and falls prevention strategies and confidence and motivation to engage in falls prevention strategies in the first month after hospital discharge?
2. Will providing a tailored individual education program for older patients that focuses on falls prevention after discharge in addition to usual care affect older patient's engagement in falls prevention strategies in the first month after hospital discharge?
3. Will providing a tailored individual education program for older patients that focuses on falls prevention after discharge in addition to usual care affect the rates of falls in the first month after hospital discharge?

3.2 Research aims

1. Evaluate the effect of providing a tailored multimedia falls prevention education program delivered by a health professional in hospital prior to discharge and in addition to usual care, on

a) Participants' self perceived risk of falls, knowledge about falls and falls prevention strategies and confidence and motivation to engage in falls prevention strategies in the first month after hospital discharge.

b) Participants' levels of engagement in falls prevention strategies in the first month after hospital discharge.

c) Number of falls and falls injuries in the first month after hospital discharge.

2. Evaluate participants' ability to engage with the education materials including auditory and visual presentation, equipment settings of the DVD and pictorial content, language, layout and readability of the written workbook.

4. Design

Randomised controlled trial (n=50)

Single blinded (baseline and outcome assessors blinded to group allocation)

Two groups – intervention and control, parallel design

- Active control group – continue with usual care
- Intervention group – receive intervention in addition to usual care

---

5. Participants and Setting

5.1 Participants

The population of interest is those older patients who are to be discharged after a hospital stay of longer than approximately 5 days. Previous studies demonstrated adverse outcomes in medical patients with a LOS of approximately 7 days[18] and our own study had an average LOS of approximately 26 days.[1] Surgical patients are excluded from this study due to the specific nature of complications associated with surgery. Although our previous observational study included patients with surgical diagnoses, we hypothesise that education may need to be specifically designed for this patient group.

Only patients who are to be discharged to the community will be included in the target population as those patients who are discharged supported accommodation are unable to engage in the decision making that this education package will suggest, such as initiating requests for home help or visiting their family doctor. Additionally research meta-analyses demonstrate that falls in supported accommodation require different interventions to those in the community.[19] We will only target patients with normal cognition because our previous large RCT (n=1206) demonstrated that education is effective when given to patients with intact cognition but education given to patients with impaired cognition results in an increased rate of injurious falls.[11]

Since telephone follow up is essential to the education intervention and measurement of the primary outcomes, participants who have sensory impairments which mean they are unable to engage with the research process through telephone call will be excluded from the study.

5.2 Inclusion Criteria

Age range: greater than 60 years old, no upper age limit, patient to be discharged within 1 week of enrolment into the study, discharged to community dwelling abode, able to provide informed, written consent, speaks English as first language.

5.3 Exclusion criteria

Discharged to hostel or nursing home, current or past diagnosis of confusion or cognitive impairment, patient is under the care of a palliative care or mental health medical officer, patient has been re-admitted to hospital within last 2 weeks, patient has hearing impairment that precludes communication via face to face discussion or telephone call. Cognitive impairment will be defined as any patients with a documented medical history of dementia or documentation of delirium or acute confusion during the hospital admission. Patients' self-report of confusion, memory loss or reported inability to participate in an education process will also be exclusion criteria. If any doubt exists about a patient's cognitive ability, the patient will be excluded from enrolment (see also section 10; Recruitment). The exclusion criteria have been developed based on our previous study which demonstrated that providing individual falls prevention education to patients with trained health professional follow up results in a significant reduction in falls in those patients with intact cognition but results in an increased rate of injurious falls in hospital in patients with impaired cogntion.[11]

5.4 Setting

The setting is the medical and rehabilitation wards of Swan Districts hospital. The rehabilitation and stroke unit is a 24 bed unit that manages older patient rehabilitation for orthopaedic and neurological conditions and general functional decline, including patients with a primary diagnosis of stroke. The population is almost exclusively over 65 years of age. The medical ward contains 48 general beds. The most common admission diagnoses on the medical ward are respiratory diagnoses such as COPD and emphysema and cardiac diagnoses such as right heart failure. Both settings provide 24 hour medical and nursing

care with access to acute diagnostic procedures. Allied health services including physiotherapy and occupational therapy are provided each week day, with weekend therapy cover provided in both wards for patients with acute medical requirements. Local falls prevention programs operate on all wards and staff receive falls prevention education as part of regular staff training programs. Patients receive falls prevention education as part of usual medical care, this is provided during the inpatient stay by allied health, medical and nursing staff and is ongoing to the patients individual medical requirements. Discharge programs at SDH are provided as required for patients and include home visiting by health care workers, home assistance and ongoing outpatient rehabilitation. In addition, diagnostic specific falls prevention measures and other relevant health information is provided for patients and their families by means of brochures and self-help guides on the ward foyer and out patients' area.

Older patients with specific mental health diagnoses are not managed on the rehabilitation or medical wards but admitted to a separate mental health unit and older patients who undergo surgical procedures are admitted to the 40 bed surgical ward. Maternity and children's wards also form part of the hospital setting. All these wards are excluded from the recruitment procedure.

---

6. Outcome Measures

6.1 Primary outcome measures

1. Participants' self-perceived risk of falls and knowledge about falls and falls prevention strategies prior to and after the education intervention, measured with a face to face survey conducted in hospital.
2. Participants' confidence and motivation to engage in falls prevention strategies in the first month after discharge, measured with a face to face survey conducted in hospital.
3. Participants' engagement in falls prevention strategies in the first month after hospital discharge measured with a custom designed telephone survey.

The primary outcomes will be measured with custom designed surveys (see attachments). These surveys are based on the constructs of the Health Belief Model of health behaviour change[20] and determine whether the education materials differentially address the four specific aims of providing the education (see section 7; Intervention). Survey items take two forms; those asking participants if they now feel more aware of these issues and those posed as 'knowledge' items, which reflected the information presented. Self-perceived awareness items are Likert scaled (strongly agree to strongly disagree). Engagement in falls prevention strategies are measured using items based on the current evidence based practice for falls prevention in the community, including our own data on the risk factors for falls in the post discharge period.[1,10, 21]

6.2 Secondary outcome measures[a]

1. Falls in the first month after discharge

The definition of a fall event will be the World Health Organisation definition namely: "an event which results in a person coming to rest inadvertently on the ground or floor or other lower level".[22]

Falls outcomes will be measured in two ways. First, a falls diary will be issued to participants at discharge (see attachment). The research assistant explains the definition of a fall as described above and asks participants to record any fall in their diary. Second the research assistant calls each participant at one month after discharge and asks about falls events using the recommended questioning method: ''In the past month, have you had any fall including a slip or trip in which you lost your balance and landed on the floor or ground or lower level?''[22] At this time participants are also asked to check their diary and to recall any events in the past month as monthly recall has been shown to improve accuracy of recall of falls events by older people.[23].

All falls events will be coded using the following descriptors: time of day, location, witnessed or unwitnessed, injury or no injury, type of injury if any, medical or no medical attention and type of medical attention, hospital admission and the amount of medical attention.

2. Clarity of the education materials

The auditory and visual presentation, equipment settings of the DVD and pictorial content, language, layout and readability of the written workbook, measured with a custom designed survey based on our previous study which also tested these outcomes.[15]

[a]Footnote: Falls and falls injuries will be the primary end point of a planned larger RCT that will have the statistical power to detect the effect of the education package on the primary outcome of falls.

7. Intervention

The intervention design features are based on our previous successful inpatient education intervention for older patients.[15] The intervention is based on pedagogically sound processes of adult learning principles [24] and is also adapted as recommended for low functional health literacy.[25] Lower levels of health literacy are more common in older people.[25] The workbook design follows the recommended principles of design for written patient education materials.[26]

The DVD and workbook are designed to contain identical content. The content depicts an older person as the patient model. The content is based on the Health Belief Model (HBM) framework for understanding health-related behaviours.[20] Examination of patient behaviour following education is a well-developed concept in the area of behavioural sciences and the HBM is one of the most widely used frameworks for predicting preventative health behaviours.[20]

The content addresses falls prevention and the aims specifically are to;

1. inform patients of the risk of falls and fall-related harm such that patients have an accurate perception of the risks they face,
2. inform patients of falls prevention strategies that they could undertake in the period after hospital discharge,
3. foster patient belief that they could successfully undertake falls prevention strategies and that if undertaken, their risk of falling will reduce,
4. provide a cue for action by facilitating patient planning to undertake falls prevention strategies. Information presented under aims i) and ii) is based upon local data and data presented in our previous research.[1,9,10,21]

The workbook and DVD are only presented in English and are not adapted for patients from a different cultural background who do not speak English as a first language. For this pilot

study only patients who speak English as a first language are eligible for inclusion in the study.

Participants receive the falls prevention education privately by their bedside, first by DVD with subsequent follow up by written delivery and are allowed to view the DVD more than once as desired and to study and keep the workbook. Participants in shared rooms received the education and survey administration at separate times with privacy screens in place. The educator facilitates optimal engagement with the education by adjusting environmental or individual elements, including seated position and application of visual or hearing aids.

The educator then discusses the education content with the participant in a manner which allows the participant to internalise the principles of the education and then develop personal falls prevention strategies through SMART goals (Specific, Measureable, Achievable, Realistic and Timely). Cues to action whereby participants understand when to engage in their strategies are also developed. Participants are encouraged to write down their goals in their workbook. We envisage that between 2 and 4 meetings will occur and most usually one meeting per day. Each meeting time is estimated to be a maximum of 15 minutes but is more likely to be between 6 to 10 minutes. Previous education intervention found that the educator spent a total (all sessions included) median time of 25 minutes per patient.[11] This included setting up the multimedia component of the intervention.

One week after participants leave hospital those participants in the intervention group receive a telephone call from the educator, which uses active learning principles to personally reinforce the contents of the education for the participant. The time estimated for the telephone call is 15 minutes but the more likely time is between 5 and 10 minutes.

---

8. Statistical Analysis

This is a pilot study. The primary purpose of the study is to measure secondary outcomes of the education intervention. This is done by measuring the efficacy of the education materials in four domains: raising the participants' self-perceived risk of the threat of falls, knowledge about falls prevention, their self-efficacy to engage in falls prevention activities and their subsequent levels of engagement in falls prevention strategies.[20] The results of this trial will inform the design and sample size calculations for a larger randomised controlled trial, which will examine the effect of the education on the primary outcome of falls after discharge.

Thematic analysis of qualitative data will be conducted to examine participants' acceptance of the education messages and their suggestions for modifications of the education presentation. In particular we will examine the effect of the education on participants' willingness to accept assistance with their activities of daily living and their engagement in health-referred exercise programs. This is based on our previous published data which identify the association between receiving assistance with activities of daily living and falls in the post discharge period and also identify barriers that older people report prevent them from engaging in exercise after hospital discharge.[1],[10]

Comparison of outcomes with ordinal scaling (self-perceived risk of falls, knowledge of falls,) between the intervention and the control group will conducted using Wilcoxon rank sum tests. Logistic regression analyses will then be conducted to determine whether the proportion of respondents who provide desired responses is different between the two groups before and after the education. Comparison of outcomes within the intervention group pre and post education will be measured using logistic regression analysis. The differences between groups of the proportions of participants engaging in falls prevention strategies after discharge will be measured using logistic regression analysis.

This study is not powered to find significant difference in falls rates but we will still complete the following analyses and examine whether our data distribution are appropriate for them.

Falls rates in the month after discharge will be analysed using Cox semi-parametric proportional hazards regression analysis (Anderson-Gill model for recurrent events). The proportion of participants having one or more falls in each group (being a faller) will be compared between groups using logistic regression.[16,17]

Responses to the survey (Likert scale data) measuring the response to the presentation of the education, such as clarity and audibility, will be analysed by generating descriptive statistics including the median and interquartile range. These analyses were conducted during our previous education trial of similar materials.[15] All analyses will be conducted on an intention to treat principle. Data management and analysis will be completed using Stata version 11.0 software (StataCorp, Texas).

We will collect data on aspects of trial procedure, including recruitment rate, percentage of drop outs from the study and will also examine whether the blinding of the research assistant is successful in the ward environment and also if nursing staff who are involved in discharge planning remain blinded to group allocation.

Associate Professor Terry P. Haines (Monash University) a recognised international falls expert with extensive experience in design and statistical analysis of falls data has provided guidance and will provide ongoing statistical expertise for this study.

---

9. Safety and Adverse Events

Potential risks of the actual education intervention are thought to be very low as all participants will continue to receive their usual medical care throughout the study. No risk above the risk of daily life is anticipated, although falls and resulting injuries and other adverse events as part of usual medical care, are known to occur frequently in this patient group.

All participants will continue to receive their usual medical care while in hospital and after discharge. In hospital, participants can be immediately reviewed by their medical team if any adverse event arises such as distress from responding to survey items, or an unrelated medical event that occurs while the participant is interacting with the researcher. At discharge participants are given a diary to record falls and are advised verbally to contact their doctor or a family member if they sustain a fall. The diary also explicitly informs participants to contact their doctor or family if they sustain a fall. Participants receive a phone call at the conclusion of the study (at 1 month). The research assistant who calls participants is a trained health professional and is made aware that the post discharge period is a time when adverse events may occur in older patients. The research assistant at this time explicitly advises participants to contact their doctor if they report any adverse event (falls or otherwise) that has occurred during their time of enrolment in the study. The research assistant also asks participants if they require any other person, such as a family member or carer, to be contacted if they report experiencing any kind of difficulty. The research assistant is able to immediately notify this person for the participant, if they so desire.

Participants could conceivably experience distress from responding to the survey, for example if they had a previous fall and this study reminds them of that event. Participants are encouraged verbally and in writing to discuss their proposed participation in the study with a family member or carer who can offer social and emotional support. Participants are also reminded at enrolment and at any other relevant times, such as during the education intervention or the telephone call, that they can withdraw from the study at any time

without prejudice, if they experience any emotional distress, such as from answering questions or engaging with the education materials.

In a previous RCT we demonstrated that providing multimedia education with trained health professional follow-up reduces falls in patients with intact cognition. However while the proportion of patients with impaired cognition who fell was comparable between intervention and control groups there was a higher rate of injurious falls (however no fractures) in patients with impaired cognition.[11] Therefore we have excluded patients with cognitive impairment from this study (see section 5; Participants and section 10; Recruitment).

10. Procedures

10.1 Recruitment

Senior nursing staff on the ward are asked to confirm that patients on the ward who have been identified by the research assistant through the ward admission codes as meeting the inclusion criteria are medically stable. The nurse asks patients if they would be agreeable to discuss their potential involvement in a study. If the patient is agreeable the research assistant then approaches the patient. Potential participants are informed both verbally and in writing of the aims and methods of the study, (see attachment) funding, institutional affiliations of the researchers, and the anticipated benefits and potential risks of the study including the time commitment of the intervention. The research assistant uses extended time where necessary to discuss the information with potential participants who have impairments that affect communication, such as hearing or visual impairments, (except hearing impairments that affect ability to engage with materials or answer a telephone survey). Participants are encouraged to discuss the information with their family member or other support person before consenting, and the research assistant is available to discuss the study with family or support persons if the participant so desires.

Once discussion is finalised with the patient and their family, and if the patient wishes to proceed with enrolment, the research assistant supervises the patient as they sign the consent form (see attachment). Participants are given a copy of the consent form to keep and the research assistant retains the signed consent form. Only patients who provide written consent will be enrolled into the study.

The research team will screen eligible participants for the presence of cognitive impairment at a number of levels during the recruitment process. Initially, after the nurse manager identifies a patient as medically stable for approaching for discussion, patient case notes are screened for any diagnosis of cognitive impairment and if there is any doubt about the information provided, medical staff are approached to clarify the information. The research assistant, who is a health professional person, will also screen patients on initial contact and if patients display any signs of cognitive impairment medical staff are approached for clarification. Any doubts about the patient's eligibility are then referred to the principal

researcher, who will discuss this with the associate researcher. Final confirmation of eligibility for enrolment will rest with the associate researcher (Assoc Prof Christopher Beer) who is a clinical geriatrician and also has extensive research experience working with older people with cognitive impairments.

10.2 Randomisation

Participants are allocated to groups in a 1:1 ratio in consecutive order after enrolment by the research assistant. The allocation is performed by the principal researcher (AM Hill) opening an opaque sealed envelope with the participant's study identification number on it. The paper inside the envelope contains the group allocation. The envelope order is determined by a computer generated random number sequence that will be produced by a research collaborator (Assoc Prof Terry P. Haines, Monash University) who is not involved in recruitment, intervention delivery or data collection. These envelopes will be posted from Monash University at the commencement of the recruitment process.

10.3 Blinding

The research assistant who enrols patients, conducts baseline assessments and one month post discharge surveys, is blinded to the group allocation throughout the study, until the final extra questions that will be asked of participants in the intervention group. These questions relate to the quality of the education intervention received and are asked at the conclusion of the post-discharge telephone survey which examines participants' engagement in falls prevention strategies in the first month after discharge. The principal researcher is not involved in baseline data collection or telephone data collection. Hospital staff who organise discharge services remain blinded to participants enrolment into the study.

10.4 Study Procedure (see attachments for surveys)

Participants enrolled (see recruitment 9.1). Demographic baseline assessment is completed by research assistant. Baseline survey that measures self-perceived falls risk and knowledge

of the nature of the falls risk and engagement in falls prevention strategies to reduce falls risk after discharge is also administered by research assistant. Participants are then randomised into two groups (see section 9.2; randomisation), the intervention and control groups. Both groups continue to receive their usual care (includes all usual discharge planning procedures). The intervention group then receive the education intervention in a pragmatic manner (see section 7; Intervention). Measurement of clarity education materials (DVD and workbook) completed by principal researcher immediately after delivery of DVD and up to 24 hours after the participant views the workbook.

Interactive sessions (2 to 4 sessions) each of 15 minutes no more than one per day, completed by principal researcher. Post education surveys of participants in both intervention and control groups administered by principal researcher. Pre-discharge survey and falls diary instruction completed by research assistant just prior to discharge.
One week post discharge education session through telephone call to participants in intervention group that reinforces the education. This call is conducted by the principal researcher. One month after discharge final discharge survey administered by the research assistant through a telephone call.

10.5 Training

The research assistant will be provided with orientation and training prior to the trial commencement. The research assistant will be a health professional person who is experienced at working with older persons in a hospital setting. Training will encompass recruitment procedures, measurement of baseline and outcome data with survey tools, data collection procedures including maintaining protocols for confidentiality of data and instruction in dealing with any adverse event that occurs during face to face or telephone contact with participants. Daily feedback and monitoring of procedure will occur in face to face meetings at the hospital and via telephone at the end of each working day (Monday to Friday).

11. **Data handling and storage**

Data from surveys will be collected on hard copy sheets. Each hard copy sheet will contain a participant number. The unique identifying code for each participant (1-50) will be entered onto a separate password protected electronic spreadsheet which is on a restricted section of the staff computer system at the University. Access to the computer storage system is restricted to staff only and the password to this spreadsheet will be known only to the researcher.

Baseline demographic data will also be entered onto handheld devices and onto hard copy data sheets. These data will be de-identified on entry – each participant will be entered as a unique number (1-50). The research assistant will transfer all data to the principal researcher at the end of each working day. The principal researcher will store data in the locked filing cabinet in a locked office. The hand held electronic data device data will be transferred onto computer spreadsheets then the hand held device will be wiped.
Data will remain de-identified on the spreadsheets when entered to the computer system

All data will be stored securely at School of Physiotherapy, The University of Notre Dame Australia in a locked filing cabinet in a restricted locked area with access only to the principal researcher. After the mandatory period for data retention all hard copy data collection forms will be confidentially shredded and securely disposed of at Notre Dame. Any electronic data on storage devices (CD) will also be disposed of in accordance with the Privacy Act 1988.

---

12. Monitoring

The principal researcher, associate researcher and research assistant will meet weekly and also maintain daily phone and email contact. The principal researcher will monitor all procedures to ensure they comply with the approved procedures.

The principal researcher will meet twice daily (once by telephone) with the research assistant to monitor research procedures, including recruitment and data collection procedures. The principal researcher will deliver the intervention, including the follow up education telephone call. The associate researcher will provide a final decision about an individual patient's enrolment if there is a query about the cognitive status of a patient. The associate researcher will also monitor the daily conduct of the trial within the site (Swan Kalamunda Health Service), by regular meetings with the principal researcher and the site sponsor (Dr Amanda Boudville).

The Principal researcher will provide ongoing reports on the conduct and progress of the trial to the Swan Districts hospital executive and to the Sir Charles Gairdner Group HREC, as well as progress reports to the School of Physiotherapy and the Research Office at The University of Notre Dame Australia. These reports will include information on progress to date, or outcome in the case of completed research, maintenance and security of records, compliance with the approved proposal and compliance with any conditions of approval.

All participants while in hospital continue to receive their usual care and supervision from their designated medical team. All participants when discharged continue to receive their usual care and supervision from discharge hospital staff, such as the visiting nurse and from their General Practitioner, who is sent a discharge summary from the hospital.

13. Ethical considerations

Conduct of this project will conform to the sentiments of the National Statement on Ethical Conduct in Human Research.[27] The researchers have made every effort to ensure that this study has merit and integrity, is respectful of participants and their families and carers or supporters and shows justice in its design. Review of current knowledge about falls prevention in post discharge populations strongly suggests that this proposal has potential benefits which outweigh the risks to participants.

Specific considerations regarding recruitment, inclusion and exclusion criteria, safety, confidentiality, informed consent and data monitoring and storage are outlined in the previous sections (see index; page 3).

13.1 Potential conflict of interest

The researchers and research assistant are not part of the patients' medical teams at the hospital site and neither the principal researcher nor the research assistant work at the hospital site or would be in a position where they would provide any medical care to potential participants. The associate researcher is employed at the hospital as a senior geriatrician but does not work on the recruitment wards and will not be involved in recruitment of patients, data collection or delivery of the intervention.

14. <u>Budget</u>

14.1 Budget

1. Materials: DVD and workbook, diaries, survey tools (50 copies, 3 DVDs) – $6,000 (Note: Portable DVD players previously purchased and exclusively available for duration of intervention)
2. Research assistant (1FTE 10 weeks, level 5.3, estimated that assistant will be employed at 0.8FTE for 10 weeks and 0.4FTE for 5 weeks, includes on-costs to the university) $14,030
3. Telephone follow – up completed from School of Physiotherapy, researcher's office.

14.2 Grant administration

Research Office
The University of Notre Dame Australia
19 Mouat St (Box 1225)
Fremantle WA 6959
Email: research@nd.edu.au
Ph: +61 8 9433 0943
Fax: +61 8 9433 0544

14.3 Contact for enquiries

Dr Anne-Marie Hill **OR**
Ms Lorraine Mayhew
Senior Administration Officer, Research Office

14.4 Grant funding (total $25,000)

The Menzies Foundation

Contact: General Manager,

The Menzies Foundation

210 Clarendon Street,

EAST MELBOURNE VIC 3002.

Email: menzies@vicnet.net.au

---

15. References

1. Hill AM, Hoffmann T, McPhail S, Beer C, Hill KD, Oliver D, Brauer SG, Haines TP. (2011). Evaluation of the sustained effect of inpatient falls prevention education and predictors of falls after hospital discharge—follow-up to a randomized controlled trial. *J Gerontol A Biol Sci Med Sci* 66A (9): 1001-1012.
2. Wolinsky FD, et al.(2009). Recent hospitalization and the risk of hip fracture among older Americans, *J Gerontol A Biol Sci Med Sci*;64:249-255.
3. Witherington EMA, et al. (2008). Communication gaps and readmissions to hospital for patients aged 75 years and older: observational study, *Qual Saf Health Care*;17:71-75.
4. Forster AJ, et al. (2003). The incidence and severity of adverse events affecting patients after discharge from the hospital, *Ann Intern Med*;138:161-167.
5. Mistiaen P, et al. (2007). Interventions aimed at reducing problems in adult patients discharged from hospital to home: a systematic meta-review, *BMC Health Serv Res*;7:47.
6. Clemson, L., et al. (2008). Environmental interventions to prevent falls in community-dwelling older people: a meta-analysis of randomized trials. *J Aging Health, 20*(954-971).
7. Bischoff-Ferrari HA, et al. (2010). Effect of high-dosage cholecalciferol and extended physiotherapy on complications after hip fracture: a randomized controlled trial, *Arch Intern Med*;170:813-820.
8. Bunn F, et al. (2008). A systematic review of older people's perceptions of facilitators and barriers to participation in falls-prevention interventions, *Ageing Soc*;28:449-472.
9. Hill AM, Hoffmann T, Beer C, McPhail S, Hill KD, Oliver D, Brauer SG, Haines TP. (2011). Falls after discharge from hospital: is there a gap between older peoples' knowledge about falls prevention strategies and the research evidence? *Gerontologist*;doi: 10.1093/geront/GNR052.
10. Hill, AM, Hoffmann T, McPhail S, Beer C, Hill KD, Brauer SG, & Haines TP. (2011). Factors associated with older patients' engagement in exercise after hospital discharge. *Arch Phys Med Rehabil*; doi:10.1016/j.apmr.2011.04.009.

11. Haines TP, Hill AM, Hill KD, McPhail S, Oliver D, Brauer SG, Hoffmann T, Beer C. (2010). Patient education to prevent falls among older hospital inpatients: a randomized controlled trial. *Arch Intern Med*;171(6):516-24.
12. Bradley C, Harrison J (2007). Hospitalisations due to falls in older people, Australia, 2003–04. *Injury Research and Statistics Series, number 32*, AIHW, Adelaide.
13. Heinrich S, et al. (2010). Cost of falls in old age: a systematic review, *Osteoporos Int*;21:891-902.
14. Anderson GF, Steinberg EP. (1984). Hospital readmissions in the Medicare population, *N Engl J Med*;311:1349-1353.
15. Hill AM, McPhail S, Hoffmann T, Hill K, Oliver D, Beer C, Brauer S & Haines TP. (2009). A randomized trial comparing digital video disc with written delivery of falls prevention education for older patients in hospital. *J American Geriatr Soc.*; 57(8): 1458-63.
16. Haines TP, Hill AM. (2011). Inconsistent results in meta-analyses for the prevention of falls are found between study-level data and patient-level data. *J Clin Epidemiol.*;64: 154-162.
17. Robertson MC et al. (2005). Statistical analysis of efficacy in falls prevention trials. *J Gerontol A Biol Sci Med Sci.*;60: 530-534.
18. Mahoney JE et al. (2000). Temporal association between hospitalization and rate of falls after discharge. *Arch. Intern. Med.* 160, 2788-2795.
19. Cameron ID et al. (2010). Interventions for preventing falls in older people in nursing care facilities and hospitals. *Cochrane Database Syst Rev*; CD005465.
20. Rosenstock IM et al. (1988). Social learning theory and the Health Belief Model. *Health Educ Behav*; 15(2): 175-83.
21. Gillespie LD et al. (2009). Interventions for preventing falls in older people living in the community. *Cochrane Database Syst Rev*; CD007146.
22. Lamb SE et al (2005). Development of a common outcome data set for fall injury prevention trials: The Prevention of Falls Network Europe consensus. *J Am Geriatr Soc;* 53(9): 1618-22.
23. Campbell AJ et al. (1997). Randomised controlled trial of a general practice programme of home based exercise to prevent falls in elderly women. *BMJ*; 315(7115): 1065-9.

24. Merriam SB, Caffarella RS.(1999). Learning in adulthood: a comprehensive guide. San Francisco: Jossey-Bass.
25. Adams RJ, Appleton SL, Hill CL, Dodd M, Findlay C, Wilson DH. (2009). Risks associated with low functional health literacy in an Australian population. *Med J Aust*; 191(10): 530-4.
26. McKenna K & Tooth L. (2006). Client education: a partnership approach for health practitioners, Sydney: UNSW Press.
27. National Health and Medical Research Council. (2007). National statement on ethical conduct in human research. Available at: http://www.nhmrc.gov.au/guidelines/publications/e72
